# Optical Coherence Tomography Investigations and Modeling of the Sintering of Ceramic Crowns

**DOI:** 10.3390/ma12060947

**Published:** 2019-03-21

**Authors:** Virgil-Florin Duma, Cosmin Sinescu, Adrian Bradu, Adrian Podoleanu

**Affiliations:** 1 3OM Optomechatronics Group, Faculty of Engineering, Aurel Vlaicu University of Arad, 77 Revolutiei Ave., 310130 Arad, Romania; 2 Doctoral School, Polytechnic University of Timisoara, 1 Mihai Viteazu Ave., 300222 Timisoara, Romania; 3 School of Dental Medicine, Victor Babes University of Medicine and Pharmacy of Timisoara, 2A Eftimie Murgu Place, 300070 Timisoara, Romania; 4 Applied Optics Group, School of Physics, University of Kent, Canterbury CT2 7NR, UK; a.bradu@kent.ac.uk (A.B.); a.g.h.podoleanu@kent.ac.uk (A.P.)

**Keywords:** Sintering, dental prosthesis, pressed ceramics, Optical Coherence Tomography (OCT), non-destructive testing (NDT), calibration of dental ovens, modeling, multi-parametric analysis

## Abstract

Dental prostheses are sintered in ovens that sometimes suffer from a loss of calibration. This can lead to variations of the sintering temperature outside the range recommended by the manufacturer. Stress and even fractures in dental ceramics may occur, and this leads to the necessity to rebuild the dental construct. The aim of this work is to monitor the quality of sintering processes using an established biomedical imaging technique—optical coherence tomography (OCT). Conventional current procedures imply the fabrication of supplemental samples that add to the expenses and are only evaluated visually. To our knowledge, we were the first to propose the use of OCT, a non-destructive method that brings objectivity for such assessments, focusing, in a previous study, on metal ceramic dental prostheses. Here, a different material, pressed ceramics, is considered, while we propose a quantitative assessment of the results—using reflectivity profiles of en-face (i.e., constant-depth) OCT images of sintered samples. The results for both the pressed ceramics and metal ceramics prostheses are discussed by obtaining the analytic functions of their reflectivity profiles. A multi-parametric analysis demonstrates the best parameter to characterize the loss of calibration of dental ovens. Rules-of-thumb are extracted; producing dental prostheses with defects can thus be avoided.

## 1. Introduction

Integral ceramics represent the ultimate level in current esthetic dentistry [1,2,3,4]. However, their final prosthetic and esthetic success is affected by different issues, related both to the material properties and to their manufacturing technology.

Dental ceramics are based on a silica network and on potash feldspar, soda feldspar, or both. To control aspects like their coefficient of thermal expansion (CTE), their solubility, and their fusing and sintering temperatures, different elements are added, such as pigments (to produce different hues), opacifiers (white-colored oxide to decrease translucency), and glasses [5]. A hot-pressed ceramic fabrication technique was introduced in the late 1980s, allowing the dental technician to create a restoration in wax (by pressing a plasticized ceramic ingot into a heated investment mold). Ceramics containing high amounts of leucite glass or optimal pressed ceramics have been initially used for this process [3], but since 2006, lithium disilicate has been the material of choice [4]. For the latter, the commonly used technique involves waxing the restoration to the full contour and then hot pressing it to yield a restoration.

Despite the growing usage of veneering porcelain to zirconia or other ceramic core materials in prosthetic constructs, clinical studies have reported veneering failure [6,7,8]. The clinical outcome relies on ceramic fractures and chipping. Most fractures start from an included defect, such as an aeric bubble, which could be inserted during the veneering of the ceramic or zirconia core with ceramic layers. Chipping is as a result of a mechanical overloading of the prosthetic construct, because ceramics are brittle; it is easy to develop when the ceramic structure has been affected in its crystalline morphology.

One of the major causes of such undesired incidents is related to the loss of calibration of the ovens used for ceramic sintering. As reported in the literature [9], and also from our own experience, after two to three years of use, there is a mismatch between the actual temperature at which the ceramic ovens are heated and the internal temperature displayed by the instrument. This issue can go undetected for long periods of time, leading to defective treatments of tenths or hundreds of dental prostheses each day. Such a loss of calibration may negatively impact numerous prostheses, and therefore numerous patients. It is thus also a source of financial and prestige loss for the dentist. A loss of calibration of dental ovens is therefore not only a research issue; a study on monitoring the temperature set in the ovens has a clinical impact.

There is no modality for a dental technician to verify that the internal temperature of the oven, where prostheses are processed, is that which has been programmed by using the sintering chart. Maintaining a well-calibrated oven over time is an issue for practitioners, as it is difficult to evaluate when calibration is needed and how often should be repeated. Ceramists complete an oven calibration at least once every six months [9], according to manufacturers’ recommendations. Only some (more expensive) ovens, such as the VITA ones, start a calibration process every time they are turned on [10]. Also, when issues are noticed, dental oven manufacturers recommend checking their calibration by fabricating supplemental samples using the clearest porcelain powder. This procedure has the disadvantage of wasting valuable materials, and thus increasing the costs. Also, the texture and translucency of the sintered ceramic is evaluated entirely subjective, as it is only performed visually. 

Objective investigation methods of the differences introduced by temperature variations in the structure of sintered ceramics may include scanning electron microscopy (SEM) and micro-CT. However, SEM requires the sectioning of the material, a process that can introduce artifacts from the very beginning of the investigations. Micro-CT involves high costs and is not currently available to dentists. Other methods, such as radiography or cone beam computer tomography, which are widely available, do not have a sufficient spatial resolution to be able to evaluate the granulation of dental ceramic materials to the level demanded, by a correct assessment of temperature variations in sintering ovens. This issue of dental sintering assessment has been approached in a previous work [11]. As far as the authors are aware, this was the first report employing optical coherence tomography (OCT) [12,13,14,15], to non-destructively and objectively evaluate the temperature variations inside dental ovens. The method proposed employs prostheses that are only currently produced, and does not require supplemental samples. This type of investigation comes in the context where the OCT method, although initially developed for eye inspection, has seen a continuous expansion to other fields, other than ophthalmology and optometry. Numerous OCT reports were published, dealing with the investigation of other types of tissues, including soft and hard tissue in the oral cavity [16], such as teeth [17,18]; interfaces with ceramic inlays [19,20]; and dental materials and constructs [21,22,23,24,25]. OCT finds also more applications in non-destructive testing (NDT), as it is used in the characterization of an increasing range of materials and applications [23,24,25,26,27,28,29].

For the present study, an in-house developed swept source (SS) OCT system was used, for its superior sensitivity [13,30] and its capability for the direct delivery of en-face images, based on the Master Slave (MS) OCT method [31]. En-face OCT images represent maps at constant depths inside the sample. In the microscopy, their orientation is more familiar than that of cross section imaging, offering an easier inspection of the granulation patterns.

While the previous report [11] was focused on metal–ceramics tooth prostheses, the first aim of our present work is to investigate the way the structure of the most modern pressed ceramic crowns changes with the temperature variations inside the sintering oven. Another aim is to model the process quantitatively, using the reflectivity profiles. We have already demonstrated that these can be obtained using en-face OCT images [11]. To elaborate, in the present study, a scope is to derive the analytic functions that can be used to describe the reflectivity variation due to the non-homogeneity in the sample granulation—in order to be able to perform a multi-parametric analysis. These functions have to be obtained for the normal sintering temperature (prescribed by the manufacturer), as well as for lower and higher sintering temperatures, corresponding to the practical limits that can be reached as a result of the loss of calibration of the dental ovens. 

To obtain a comprehensive and rigorous analysis, data from our previous study are also considered in this respect [11], for a different material with regard to the present study, focused on pressed ceramics. The scope is to consider the reflectivity profiles and their analytic functions, as well as to explore all of the possible parameters that can be derived from them (for both ceramic materials), in order to determine the most appropriate parameters so as to characterize the modification of the granulation due to a change in the sintering temperature. A final aim of the present study is thus to provide a methodology (with one or more characteristic parameters) to guide oven users. Rules-of-thumb are sought that can be applied on the reflectivity profiles obtained from en-face OCT images, in order to be used to evaluate such a loss of calibration.

## 2. Materials and Methods

### 2.1. Samples Preparation

The material employed in this study is Ceramco iC Integrated Ceramics (Dentsply International, Inc., York, PA, USA) with a CTE of 13.0 (µm/m·K) (for temperatures of 20 to 500 °C), a heating rate of 60 °C/min, flexural strength of 130 MPa, and leucite grain sizes of 1 to 5 µm [32]. We choose Ceramco iC because it is an integrated ceramic system that offers a single set of porcelains, pressing materials, and stains—for single or multi-unit porcelain-fused-to-metal and press-to-metal restorations, as well as for all ceramics, including crowns, veneers, inlays, and on-lays. It provides a high-quality in-shade consistency, and therefore ensures a reliable, life-like shade match on combination cases involving more than one type of substructure. Because of these advantages, dental technicians do not need to store other ceramic systems for different technologies. The system includes one color correlated shade system with 16 A–D shades, 26 CC Shade Series, and 4 bleach shades; it ensures consistency and esthetic quality across the different possible combination cases, and it also allows for grouping work and reducing processing time [32]. 

Thirty dental crowns made from pressed ceramics have been used for this study. They have been divided into three groups, corresponding to three types of firing procedures, namely: 

**Group L**: with samples represented by crowns pressed at 840 °C, that is, 50 °C below the normal temperature of 890 °C prescribed by the manufacturer for these ceramics.

**Group N**: with samples represented by crowns pressed at the normal temperature of 890 °C. 

**Group H**: with samples represented by crowns pressed at 940 °C, that is, 50 °C above the normal temperature. The choice of these temperature limits is based on our experience regarding the possible extreme variations of the sintering temperature inside the dental oven. 

**Figure 1:**
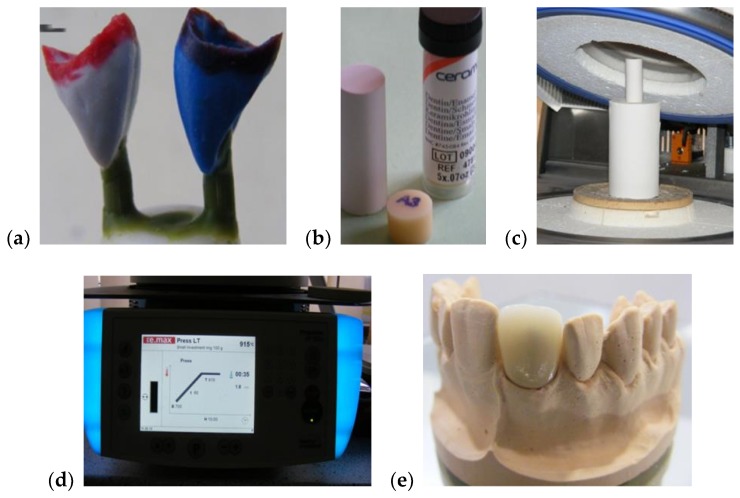
Aspects from the samples preparation: (**a**) making of the wax ups; (**b**) pressed ingots used in the study; (**c**) positioning of dental material in the oven in order to press the ceramic ingot; (**d**) ceramic pressing procedure; (**e**) example of a final dental ceramic crown.

The main steps of the manufacturing process are pointed out in Figure 1. A class 4 dental stone was prepared for pouring the model cast for each considered sample. Two coats of die spacer, to within 1.0 mm of the prepared margin, were applied for all of the samples. This served to provide 0.1 mm of clearance for the resin cement and compensated for the undercuts. In the following step, the wax-up procedure was initiated. To completely cover the restoration, a wax layer with a thickness of at least 0.8 mm was used. Then, sprues of 3 mm in diameter and 4 mm in length were used for spruing the wax-up. For each sample, a single sprue was attached directly to each incisal edge of the wax up. A clearance of 8 mm was allowed between the topmost point of the wax up and the leveling ring. 

To obtain a correct expansion, DENTSPLY Prosthetics Press Investment Powder (York, PA, USA) was used for the investing procedure in the proportion of 22 mL of liquid at 3 mL of distilled water. The liquid was added to a slightly moistened mixing bowl, followed by the powder, then hand-mixed for 15 s, and then processed for 60 s in a vacuum mixer. Each sample was carefully painted with the investing, and the remaining investment material was poured into the silicone ring. Finally, the investment was allowed to bench-set for at least 20 min. These crowns were pressed using one of the more opaque dentin shaded ingots. The pressing procedure followed several steps, namely: a low temperature of 700 °C, heat rate of 60 °C per min., high (normal) temperature of 890 °C, hold time of 20 min, and pressing time of 20 min.

A second type of dental prosthesis, metal ceramic, approached in a previous study [11], was also considered in the present one—in order to utilize the data obtained in the literature [11] for the multi-parameter analysis in Section 4. Duceram Kiss ceramics (DeguDent GmbH (a Dentsply Sirona Company), Hanau-Wolfgang, Germany) was employed for these metal ceramic prostheses—using the characteristics and sample preparation described in the literature [11]. Five groups were considered in the OCT imaging of these metal ceramic prostheses, namely: Group L100, for which the ceramic layers were sintered at 830 °C (100 °C below the normal temperature of 930 °C, recommended by the manufacturer mentioned above); Group L30, for which the ceramic layers were sintered 30 °C below the normal temperature; Group N, for which the ceramic layers were sintered at the normal temperature of 930 °C; Group H30, with ceramic layers sintered 30 °C above the normal temperature; and Group H50, with ceramic layers sintered 50 °C above the normal temperature [11]. Referring to both types of dental prostheses above, all of the samples were cooled down in the same conditions, stored in special boxes, and marked and prepared for the OCT non-invasive evaluations. To fully comply with the manufacturers’ requirements (or to the simulated errors of the oven), and to minimize human errors, the technician’s work was continuously supervised during the manufacturing phases.

### 2.2. In-House Developed MS/SS-OCT System

Optical coherence tomography was employed for the non-invasive evaluations of all of the considered samples, with the imaging processing performed using ImageJ (Version 1.52i) [33] and MATLAB (Version R2018b, The MathWorks, Inc., Natick, MA, USA). 

**Figure 2:**
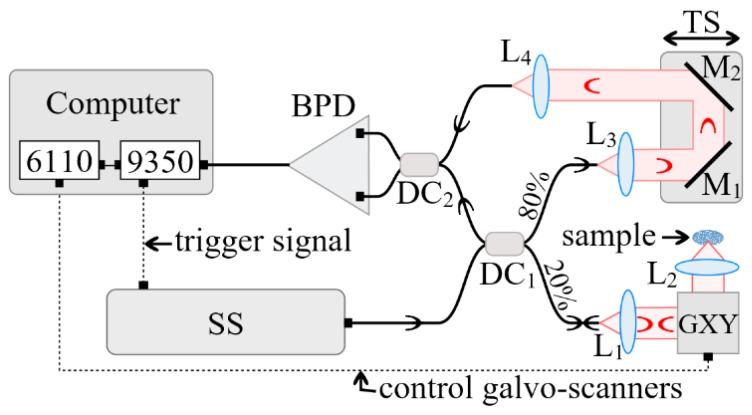
In-house developed Master Slave (MS)/swept source (SS)-optical coherence tomography (OCT) system. Components: SS—swept source; DC_1,2_ single mode directional couplers (20/80 and 50/50, respectively); GXY dual axis XY galvanometer scanner; L_1–4_, achromatic lenses; BPD—balanced photo-detector; TS—translation stage.

A schematic diagram of the in-house developed MS enhanced SS-OCT imaging system utilized in the study is presented in Figure 2. A swept source (SS) laser (Axsun Technologies, Billerica, MA, USA) with a central wavelength of 1060 nm, a sweeping range of 106 nm (quoted at 10 dB), and a 100 kHz sweeping speed was employed. The minimum reflectivity measurable by the system was determined by its sensitivity, which, in this case, was experimentally measured as superior to 95 dB. The measured axial resolution provided by the instrument was 10 µm, measured in air [31].

The interferometer configuration employs two directional couplers, DC_1_ and DC_2_. DC_1_ has a splitting ratio of 20/80, while DC_2_ is a 50/50 balanced splitter that feeds a balance detection receiver balanced photo-detector (BPD) (Thorlabs, Newton, NJ, USA, model PDB460C). In the sample arm of the interferometer, the beam was collimated by the achromatic lens L_1_ (focal length 15 mm), and conveyed towards a scanner head GXY made of two orthogonal galvanometer scanners (Cambridge Technology, Bedford, MA, USA, model 6115) and focused on the sample by the scanning lens, L_2_, (25 mm focal length). The optical power onto the sample was 2.2 mW, while the optical components in the sample arm determined a lateral resolution of 10 µm. The reference arm consisted of two collimating lenses, L_3_ and L_4_, both similar to L_1_, as well as two flat mirrors (M_1_ and M_2_) placed on a translation stage (TS). By adjusting the position of the TS, the optical path difference (OPD) in the interferometer can be adjusted. 

The output of the photo-detector BPD feeds the electrical signal to one of the two inputs of a fast digitizer (Alazartech, Quebec, Canada, model ATS9350, 500 MB/s). The acquired channeled spectra were manipulated via in-house program software (Version 1.0) implemented in LabVIEW 2013, 64 bit, deployed on a PC. The same program was also used to drive the two orthogonal galvanometer scanners via a data acquisition board (model PCI 6110, National Instruments, Austin, TX, USA). A number of 500 × 500 channeled spectra were acquired to build a three-dimensional (3D)/volumetric OCT image and to produce, using the MS protocol [31], C-scans/en-face images. The 500 sweeps per line in the raster required 5 ms. Using a triangular waveform [34] to drive the fast scanner that determine the line in the final raster required a period of 10 ms, hence, this scanners was driven at 100 Hz. The 500 lines, that is, 500 B-scans/cross-sections for a volume of 500 × 500 lateral points, required 5 s.

### 2.3. Image Processing

The investigation of all of the cooled down samples was made on en-face OCT images. The B-scan images were used to correctly position the samples axially. The outer surface of the samples was curved and presented a different curvature shape amongst the different dental prostheses. Therefore, the outer surface cannot be used for the granulation assessments. 

**Figure 3:**
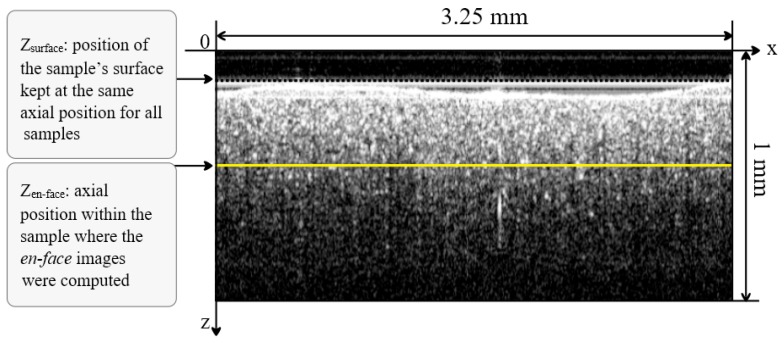
Example of an OCT B-scan/cross-section to show the method of selection of the depth from where the en-face images were selected. For all of the samples, their outer surface at *z*_surface_ was adjusted with respect to OPD = 0, to produce all of the en-face OCT images (for all samples) from a similar depth (*z*_en-face_).

For a fair comparison, the en-face images from the prostheses investigated were acquired from a similar depth (z_surface_), measured from the plane corresponding to OPD = 0 (Figure 3). All of the en-face images were acquired from a similar depth position, chosen at approximately *z*_en-face_ = 0.375 mm (distance measured in air) from the top of the vestibular surface of each sample in the B-scan image (Figure 3).

The lateral size of the en-face OCT images was *x* = *y* = 3.25 mm and was determined by the amplitude of the wave forms applied to the two galvanometer scanners. Along the in-depth *z*-axis (perpendicular on the vestibular surface), the maximum axial range determined by the sampling rate data was digitized, measured in air was *z* = 3.75 mm. By considering an estimated *n* = 1.5 refractive index for the inspected ceramics, for the central wavelength of the SS used, the axial range inside the material along the vertical axis in Figure 3 became *z*/*n* = 2.5 mm.

The surface considered at the depth of *z*_en-face_ = 0.375 mm is capable of providing a good reflectivity signal in order to characterize the granulation in the en-face OCT image acquired from inside the material. The larger the depth, the less the signal backscattered due to the absorption and scattering in the sample. The depth of *z*_en-face_ = 0.375 mm was selected as a compromise between the loss of signal and the granulation dependence on the curvature of the top surface. We thus ensured, as the optical power on the sample was kept constant, that any change in the image brightness of the sample was only due to the changes in its optical properties, and not to the differences due to the different investigation depths inside the samples or to the shape of the teeth prostheses if the investigations were made at their (uneven) surface.

A remark must be made regarding the depth of the points sampled in the OCT en-face image. As the top surface of the samples was curved, the points in the en-face image are not from a flat surface, but from the points situated at a similar OPD inside the sample. A rigorous interpretation of the data should therefore consider the curvature of the sample top surface. 

Regarding the acquisition of en-face images, they are not rendered from the volumetric dataset, as is the case when using conventional OCT technology. They are generated in real time, directly, using the MS method detailed in the literature [31]. Here, we demonstrate that a single en-face OCT image, at a suitably selected depth, can provide all of the necessary information for the evaluation of the actual temperature reached in the dental ceramic oven.

**Figure 4:**
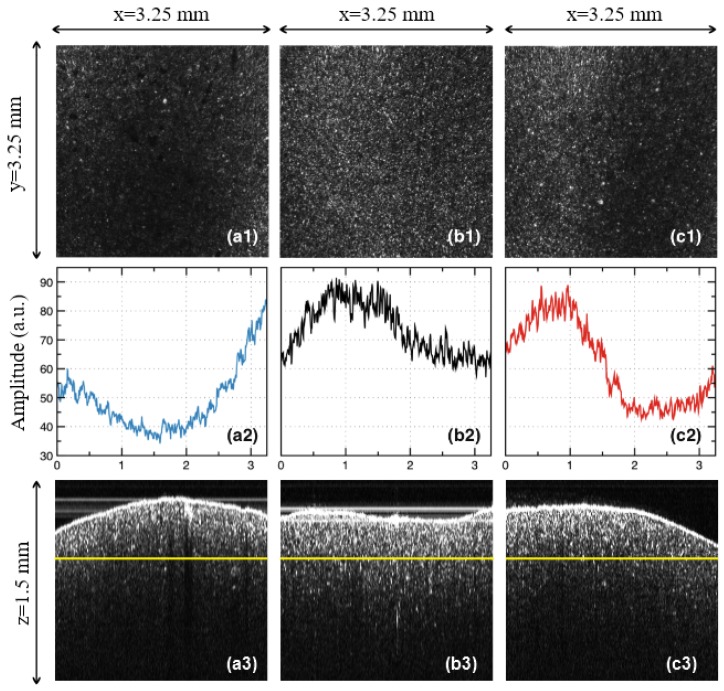
Examples of the OCT study of the samples pressed at different temperatures: (**a1**–**a3**), Group L: sample pressed at 840 °C (50 °C below the normal temperature). (**b1**–**b3**), Group N: sample pressed at the normal temperature of 890 °C, prescribed by the manufacturer. (**c1**–**c3**), Group H: sample pressed at 940 °C (50 °C above the normal temperature). Steps of the analysis: Row (**1**), en face OCT image of the sample; Row (**2**), MATLAB processing of the en face OCT image; Row (**3**), B-scan (cross-section) OCT image—with the yellow line marking the sectioning plane used to obtain the en face OCT image (taken at a constant depth in the sample, considered from the zero OPD).

In order to quantify the effect of the temperature variations inside the oven, 500 lines in the en-face OCT image from different *y* positions were averaged, and the result is displayed as a function of the reflectivity with regard to the position on the *x*-axis (Figure 4(a2,b2,c2)). In order to obtain such reflectivity profiles from en-face images, two steps are taken, namely: (i) Each original gray-scaled en-face image is converted into a binary image by replacing all of its pixels with the value of 1 (white) if the gray-scale value is above a threshold (127), while all of the other pixels are replaced with 0 (black). (ii) The white features identified in the binary image are counted to quantify the granulation, similar to the procedure described in our previous work [11]. Further on, in the present study, we employ another, more analytical approach to process the data. Such a method fulfills the aim of the present study, different from that of pure imaging, oriented towards providing a simple, fast, and practical assessment of the deviations of ovens from their best setting in terms of the sintering temperature.

As a remark, one may investigate the sample from different sides, on a number of surfaces. However, in order to develop a simple and practical method, we have made the investigation strictly beneath the vestibular surface of the sample. Similar results are expected to be obtained on any of the other sides of the prostheses. Another aspect to be highlighted refers to the orientation of the en-face images. As in can be seen in Figure 4(a1,b1,c1), they were all placed so that the dark and light bands/areas of Group L and H appeared parallel to the *y*-axis.

In this way, the profiles obtained in Figure 4(a2,b2,c2) show clearly the differences between the different areas of the sample. In order to apply the quantization developed in the following, this procedure must always be followed.

## 3. Results

### 3.1. OCT Imaging

For the three considered groups of pressed ceramics, sintered at different temperatures, the OCT evaluation revealed the following aspects:

**Group L**: samples sintered at 840 °C (i.e., 50 °C below the normal temperature prescribed by the manufacturer). In the en-face OCT image in Figure 4(a1), a decrease in reflectivity can be seen with regard to the Group N in Figure 4(b1); also, band-like shapes with alternations between more and less reflective areas can be noticed, even if the non-reflective area is wider spread. These findings can be easily processed on the images processed using MATLAB (Figure 4(b2)). 

**Group N**: with samples sintered at the normal temperature prescribed by the manufacturer, (i.e., 890 °C). From the en-face OCT image in Figure 4(b1), the reflectivity has a much more normal distribution than for the other two groups (Figure 4(a1,c1)); however, a slight alternation of more reflective with not-so reflective areas can be spotted on all of the similar samples. This can also be seen in the MATLAB processed image shown in Figure 4(b2), obtained from averaging the reflectivity values along the *y*-axis for each *x* position (as explained in Section 2.3). 

**Group H**: with samples sintered at 940 °C (i.e., 50 °C above the normal temperature). An even stronger alternation between the reflective and non-reflective areas, with a decreasing trend in width can be noticed in both the en-face OCT image (Figure 4(c1)), and in the profile obtained using MATLAB (Figure 4(c2)); also, particularly bright spots with regard to Group N are seen. Temperature affects sintering as a result of the temperature-dependence of the viscosity of silicates [35,36,37,38,39]. Thus, increasing the sintering temperature for dental porcelain leads to several consequences, namely: a decreased apparent specific density of the fired porcelain because of an increased volume; an increased total porosity; an increase of the average size of the pore; fewer but larger, and also more spherical pores, under the influence of surface tension; an increased liquid content, as bubbles in the compact expand when the viscosity decreases to a certain level by increasing the temperature; and losses of the surface detail, as there is a marked increase in the pyroplastic flow of the porcelain (thus the material may appear glassy and often takes on the greenish tinge of natural glass).

From all of the three groups, it can be concluded that the B-scan images of all of the samples are much less relevant from the point of view of the assessment of the sintering temperature variations (Figure 4(a3,b3,c3)). The same conclusion can be reached for the A-scans, that is, for the reflectivity profiles along the depth (*z*-axis). In this respect, our study demonstrated that only en-face OCT images allow for the proposed assessment. 

**Figure 5:**
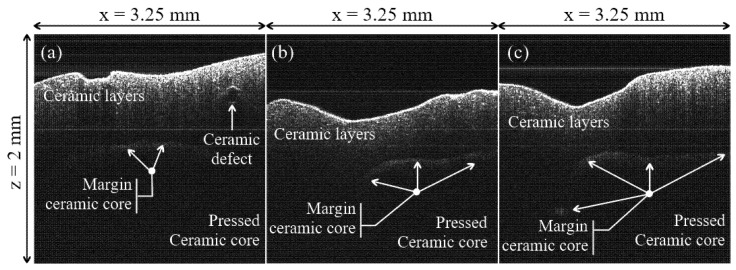
Examples of OCT B-scans/cross-sections of samples from the three groups considered in the study: (**a**) Group L; (**b**), Group N; (**c**) Group H. Material defects and margins of the pressed ceramic core can be remarked.

However, B-scans do allow for a clear identification of the ceramic pressed core, with regard to the ceramic layers that are added after them to achieve the esthetic effect. Thus, for Group N, the margin of the ceramic pressed core is normal, as seen in Figure 5b, while for Group H, this margin is better defined, as seen in Figure 5c. For Group L, the margin of the ceramic pressed core is shallower, as displayed by the image in Figure 5a. 

Other aspects are related to the ceramic layers added over the ceramic pressed core, as follows: for Group N, these layers have a relatively uniform distribution of the ceramic particles scattering between the occlusal margins, and are close to the margin of the ceramic pressed core, as shown in Figure 5b. For Group H, the scattering area is reduced, with a contraction towards the incisal margin, where the scattering is stronger, as can be seen in Figure 5c.

For Group L, the layers of the adjacent ceramic structure display an increase in the scattering of the ceramic particles, much closer to the margin of the ceramic pressed core, with defects in this area (Figure 5a). One can explain this structure from the substrate being insufficiently baked at the recommended temperature; this may provide water particles that can induce defects in this area.

### 3.2. Modeling of the OCT Reflectivity Curves—For Dental Pressed Ceramics

**Figure 6:**
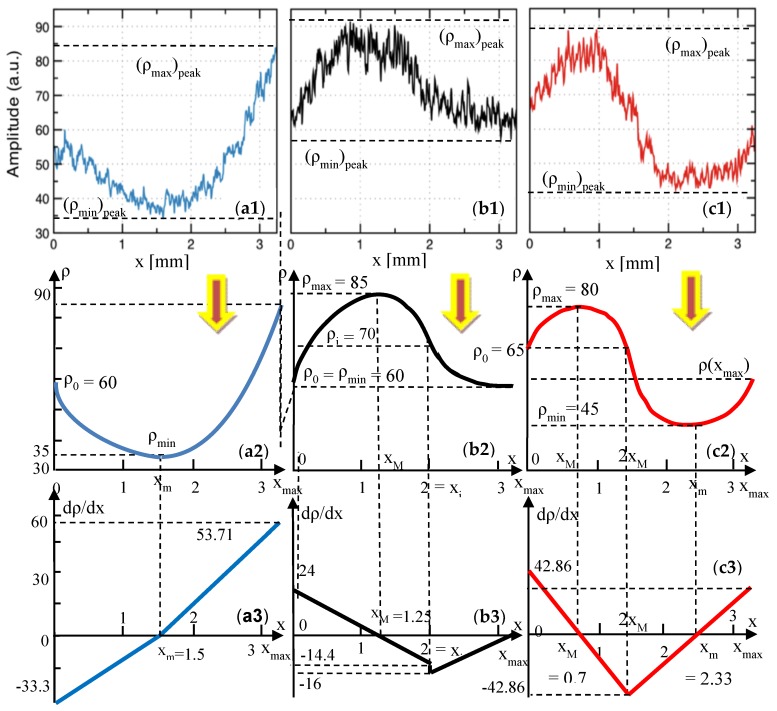
(**a1**–**a3**), reflectivity profiles obtained using MATLAB from the en-face OCT images in Figure 4—for pressed ceramic. Row (**2**): reflectivity graphs, obtained as averages of these reflectivity graphs. Row (**3**): gradient of the reflectivity, obtained from Appendix A.1.1, Appendix A.1.2 and Appendix A.1.3, as well as shown in Table 1, Table 2 and Table 3, respectively. Column (**a**), Group L: sample pressed at 840 °C (50 °C below the normal temperature). (**b1**–**b3**), Group N: sample pressed at the normal temperature of 890 °C, prescribed by the manufacturer. (**c1**–**c3**), Group H, sample pressed at 940 °C (50 °C above the normal temperature).

From the en-face OCT images in Figure 4(a1,b1,c1), the reflectivity profiles are obtained as explained in detail in Section 2.3. They are represented in Figure 4(a2,b2,c2), for each group considered for the pressed ceramics. These graphs are considered further on for processing in Figure 6(a1,b1,c1), and from each of them, a reflectivity graph *ρ*(*x*) is obtained in Figure 6(a2,b2,c2), as an average of each reflectivity profile. 

The problem is to model each graph *ρ*(*x*) as a function of the position on the *x* axis in the en-face OCT image. The easiest way to obtain these functions is to consider the parabolic portions. For each of the *ρ*(*x*) graphs, this is done in Appendix A.1.1, Appendix A.1.2 and Appendix A.1.3, for Groups L, N, and H, respectively. For each group, two parabolic functions, f(*x*) and g(*x*), were obtained on *x* intervals specific for each case, and using the values of the reflectivity *ρ* at certain positions *x* (Figure 6(a2,b2,c2)).

The analytical expressions of these two functions—and therefore of *ρ*(*x*)—are obtained for each Group, in Table 1, Table 2 and Table 3, respectively, as well as for the gradients of the reflectivity d*ρ*/d*x* and the second derivatives d^2^*ρ*/d*x*^2^. The gradients are also shown in Figure 6(a3,b3,c3), for the considered parabolic functions.

### 3.3. Modeling of the OCT Reflectivity Curves—For Metal Ceramic Prostheses

**Figure 7:**
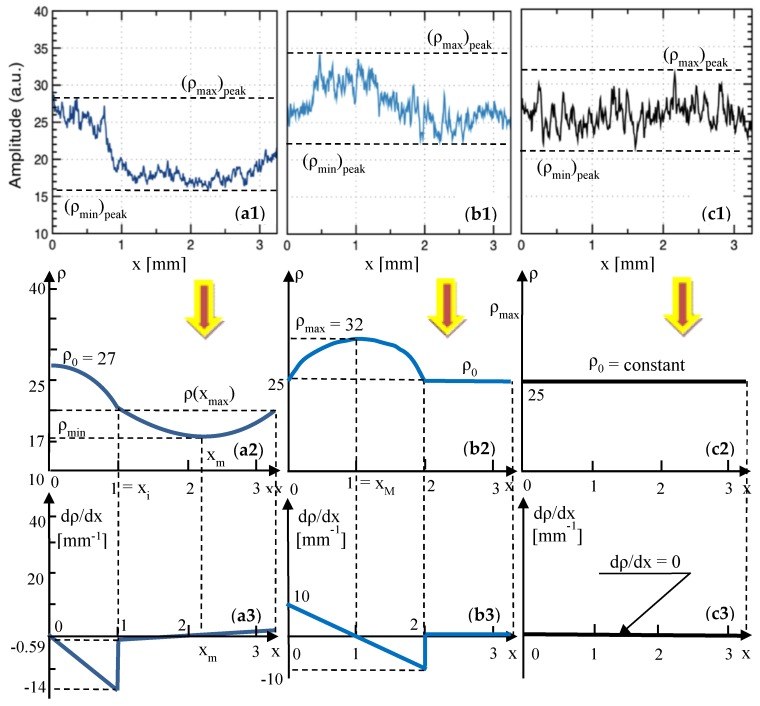
Row (**1**): reflectivity profiles extracted from our previous work [11] (reproduced with permission from MDPI) from en-face OCT images for metal ceramic dental prostheses. Rows (**2**) and (**3**): reflectivity graphs (i.e., averages of the reflectivity profiles) and gradient of the reflectivity, respectively, obtained from Appendix B.1.1 and Appendix B.1.2, and shown in Table 4 and Table 5. (**a1**–**a3**), Group L100: for the sample pressed at 830 °C (100 °C below the normal temperature). (**b1**–**b3**), Group L30: for the sample pressed at 900 °C (30 °C below the normal temperature). (**c1**–**c3**), Group N: for the sample pressed at the normal temperature of 930 °C, prescribed by the manufacturer.

**Figure 8:**
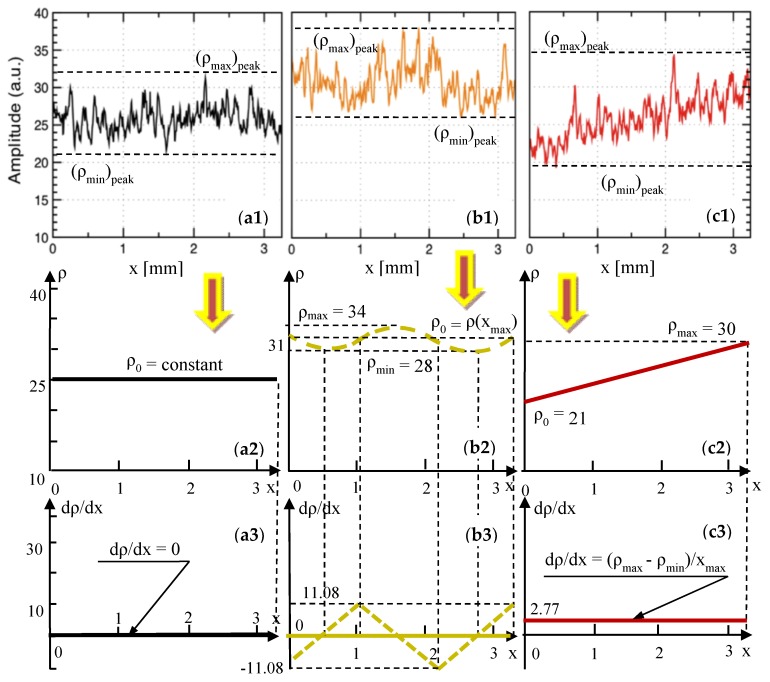
Row (**1**): reflectivity profiles, extracted from our previous work [11] (reproduced with permission from MDPI) from en-face OCT images for metal ceramic dental prostheses. Rows (**2**) and (**3**): reflectivity graphs (i.e., averages of the reflectivity profiles) and gradient of the reflectivity, respectively, obtained from Appendix B.1.3 and in the text, as well as shown in Table 6, for Group H30. (**a1**–**a3**), Group N: for the sample pressed at the normal temperature of 930 °C, prescribed by the manufacturer. (**b1**–**b3**), Group H30: for the sample pressed at 960 °C (30 °C above the normal temperature). (**c1**–**c3**), Group H50: for the sample pressed at 980 °C (50 °C above the normal temperature).

From the study on metal ceramic dental prostheses [11], the relevant reflectivity profiles have been extracted in Figure 7(a1,b1,c1), as well as in Figure 8(a1,b1,c1)—where Figure 7(c1) is identical to Figure 8(a1), as it refers to Group N of the metal ceramic prostheses. These profiles are processed in Figure 7(a2,b2,c2), as well as in Figure 8(a2,b2,c2)—where Figure 7(c2) is identical to Figure 8(a2)—and the corresponding reflectivity graphs *ρ*(*x*) are obtained. 

Further on, similar to the procedure in Section 3.2, each curve *ρ*(*x*) is modeled by considering one or two portions—parabolic, parabolic and linear, or purely linear. This was done in Appendix B.1.1 and Appendix B.1.2, for Groups L100 and L30, respectively; the results are given in Table 4 and Table 5, respectively. For each *ρ*(*x*) curve, two parabolic functions, f(*x*) and g(*x*), were obtained with analytical expressions of their coefficients, using the notations of the reflectivity *ρ* and the corresponding coordinates *x* (Figure 7(a2,b2)). Functions f(*x*) and g(*x*), as well as the gradients of the reflectivity d*ρ*/d*x* and the second derivatives d^2^*ρ*/d*x*^2^ were obtained in Table 4 and Table 5, and the gradients also in Figure 6(a3,b3). 

The case of Group N is interesting, as *ρ*(*x*) is a horizontal line, and therefore the gradient is null.

For Group H30, the *ρ*(*x*) curve has only slight oscillations, as modeled in Appendix B.1.3—see Figure 8(b2). Three parabolic functions, f(*x*), g(*x*), and h(*x*), are given in Table 6, with the analytical expressions of their coefficients, obtained using the notations in Figure 8(b2). The gradient of the reflectivity d*ρ*/d*x* and the second derivatives d^2^*ρ*/d*x*^2^ are obtained in Table 6, with the gradient also being shown in Figure 8(b3).

From Figure 8(c2), for Group H50, *ρ*(*x*) is a line, with the equation

(1) $$\rho (x)=\frac{\rho_{\max}-\rho_{0}}{x_{\max}}x+\rho_{0}=2.77x+21$$

Therefore, its gradient is d*ρ*/d*x* = constant and d^2^*ρ*/d*x*^2^ = 0.

## 4. Discussion

Based on the results obtained and on the modeling performed, a series of rules-of-thumb to complete the proposed assessment can be extracted, as follows:

- Referring to the OCT investigations:

(i) Differences in granulation between the samples sintered at different settings of the oven temperature cannot be distinguished on OCT B-scans (cross-sections), like those in Figure 4(a3,b3,c3). However, interfaces between the ceramic layers and defects can be seen on these B-scans, as pointed out in the examples in Figure 5.

(ii) In contrast, C-scans/en-face OCT images like those in Figure 4(a1,b1,c1) allow for a qualitative assessment of the above differences, which can be used to point towards temperature variations. Thus, the en-face image of the Group N sample shows a quite uniform granulation at the considered depth inside the sample. A strong reflectivity from the individual grains can be seen on the entire area, although a certain non-uniformity can be spotted—quantified further on in Figure 4(b2).

(iii) Unlike the Group N sample, the Group L and H samples (Figure 4(a2,c2), respectively) do not show a uniform granulation (and reflectivity).

(iv) Thus, images like the one in Figure 4(a1), darker that the one in Figure 4(a2), are typical for a sintering temperature lower than normal, where the ceramic material is not burned properly, and therefore the sintering is not complete. The lack of normal granulation and reflectivity in Figure 4(a1) compared with Figure 4(b1) clearly shows that.

(v) Images like those in Figure 4(c1), for pressed ceramics, are typical for the case of over burned ceramic materials, as also seen for another ceramics in the literature [11]. Indeed, one can notice band-like portions of high reflectivity—with singular bright distinctive spots, not seen in Figure 4(b1)—alternating with darker portions of lower reflectivity. The distinctive bright spot corresponds to an excessive growth of the grains, displaying a tendency to the non-homogeneity of the sample. This conclusion is in line with our previous findings in the literature [11], for another ceramics.

(vi) Relevant examples of OCT images are presented for each group in Figure 4, but similar remarks can be made for all of the samples from each group (fired at the same sintering temperature).

Such assessments were also carried out in our previous work [11] for metal ceramic prostheses, concluding from the en-face OCT imaging that the samples sintered at temperatures lower-than-normal are insufficiently baked (which lowers the mechanical resistance of the prostheses), while the samples sintered at temperatures higher-than-normal are over baked (which produce stresses and defects in the material, therefore sources of chipping). 

While the above rules-of thumb may be found useful in the assessment of the actual temperature of the dental sintering oven, they are still only qualitative. Therefore, another assessment method is discussed below.

- The reflectivity profiles in Figure 4(a2,b2,c2) are used to complete this assessment. Based on these profiles, several remarks can be made, as follows:

(i) The most “horizontal-like” profile is the one in Figure 4(b2)—for the Group N sample. This proves some relative homogeneity of the granulation in the OCT image in Figure 4(b1). However, the granulation is not perfectly uniform. In fact, some variations can be noticed, but only using this reflectivity profile, which cannot be inferred from the OCT image in Figure 4(b1) alone. One may thus conclude that such profiles can serve as a quality indicator when comparing different ceramic materials.

(ii) On the contrary, there are much higher differences between the extreme reflectivity levels in Figure 4(a2,c2)—larger for Group L with regard to Group H. Lower reflectivity values are obtained for the unbaked ceramic; in fact, almost the entire profile for the Group L sample stays at a very low level in Figure 4(a2).

(iii) The profile for the Group H sample in Figure 4(c2) also has a specific shape, roughly symmetric in the positive and negative variations around the average reflectivity in Figure 4(c1).

A similar set of remarks can be made for the reflectivity profiles of the OCT images [11] for metal ceramic prostheses in Figure 7(a1,b1,c1) and Figure 8(b1,c1), as follows:

(iv) Group N is, in this case, characterized by a reflectivity profile (Figure 7(c1) and Figure 8(a1)) that is practically horizontal, and the dispersion in reflectivity (therefore in granulation) is minimal.

(v) In contrast, as the sintering temperature is lowered (as shown in Figure 7(b1) for Group L30 and in Figure 7(a1) for Group L100) the differences in the reflectivity levels increase progressively. This can be a relevant (however, still a qualitative) indication of a lower than normal sintering temperature in the oven. Corroborated with low values of the reflectivity (as in Figure 7(a1)), this indicates a much-too-low temperature in the oven.

(vi) For higher-than-normal sintering temperatures (Figure 8(b1,c1)) a clear increase in reflectivity can be noticed, with a higher average level of the reflectivity profile for Group H30 (i.e., for a temperature increase that is not yet critical), as seen when comparing Figure 8(a1,b1).

(vii) A tilted reflectivity graph is characteristic for a much higher-than-normal sintering temperature (as it can be seen from Figure 8(c1) for Group H50). This is already correlated with high stresses in the ceramic material, which can already lead (for such high temperatures in the oven) to defects and cracks in the material, as shown in the literature [11].

Both methods of assessment discussed above, I and II, are useful in the proposed assessment, but they are still qualitative. To improve the quantitation, we used the reflectivity profiles to extract (in Figure 6, Figure 7 and Figure 8) the average curves *ρ*(*x*) of the reflectivity with regard to the *x*-position of the *y*-line (the *xy* plane being parallel to the *z*_surface_ plane (Figure 3), and thus to the plane of the en-face OCT image).

- Using the reflectivity graphs *ρ*(*x*) obtained in Figure 6, Figure 7 and Figure 8, their gradients d*ρ*/d*x*, their second derivatives d^2^*ρ*/d*x*^2^, and the parameters obtained further on in Table 7 and Table 8 (i.e., for each of the two considered ceramics), the following remarks can be made:

(i) The minimum reflectivity, both as the average value *ρ*_min_, and as the extreme one (*ρ*_min_)_peak_, clearly indicates a lower-than-normal oven temperature (Group L, Figure 6(a1,a2)). However, this indicator is not sufficient to draw a correct decision in terms of the temperature. It has to be correlated with other aspects, as for Group H, both values, *ρ*_min_ and (*ρ*_min_)_peak_ are close to those for Group N (Figure 6(b1,b2)).

(ii) The maximum values, *ρ*_max_ and (*ρ*_max_)_peak_, have little relevance to distinguish between Group N and the other groups, as can be seen from their fluctuations in both Table 7 and Table 8 (i.e., for both materials).

(iii) A more appropriate indication of a variation of the oven temperature is the difference

(2) $$\Delta \rho =\rho_{\max}-\rho_{\min}$$

which is more relevant than the difference

(3) $${\left(\Delta \rho \right)}_{peak}={\left(\rho_{\max}\right)}_{peak}-{\left(\rho_{\min}\right)}_{peak}$$

as the former is less susceptible to fluctuations. Otherwise, both differences are consistent with the temperature variation; as this variation is increased, Δ*ρ* and (Δ*ρ*)_peak_ also grow—see Table 7 and Table 8 (however, with a slight decrease for Group H50 in Table 8). In Equations (2) and (3), (*ρ*_min_)*_peak_* and (*ρ*_max_)*_peak_* were obtained by (also) considering the small, high spatial frequency variations of the reflectivity *ρ*, (i.e., the envelopes of reflectivity profiles, in Figure 6, Figure 7 and Figure 8). They can be therefore influenced by issues such as the noise or different attenuations produced by different layers of materials on top of the considered en-face image. In contrast, *ρ*_min_ and *ρ*_max_ are obtained by considering the minimum and the maximum values of the averaged graphs of *ρ*(*x*).

(iv) While Δρ is minimal for Group N (for both ceramics), it can therefore be used in the proposed assessment, and the values of this parameter are different for each material, as the levels of reflectivity are not the same. As a consequence (and also taking into account the slight lack of consistency pointed out at the previous point), a better synthetic parameter to characterize the temperature variations can be the ratio of the maximum and minimum reflectivity. We may also say, similar to the set of parameters in Equations (2) and (3), that the ratio

(4) $$k=\rho_{\max}/\rho_{\min}$$

is more appropriate, more “stable” to use than the ratio

(5) $$k_{peak}={\left(\rho_{\max}\right)}_{peak}/{\left(\rho_{\min}\right)}_{peak}$$

The value of the ratio *k* is close to 1 for Group N, for both ceramics. Such a tendency (of a *k* close to 1 and “drifting” away as the sintering temperature “drifts” away from normal/prescribed) is logical to appear in any ceramics sintering process, as a result of the (relative) homogeneity of the grains for the normal sintering temperature.

(v) The maximum gradient

(6) $${\left(d\rho /dx\right)}_{\max}=\max\left\{\left|df/dx\right|,\left|dg/dx\right|\right\}$$

of the reflectivity graphs is clearly smaller for Group N (Table 7). However, for metal ceramics prostheses (Table 8), fluctuations can be observed.

(vi) The maximum value of the second derivatives

(7) $${\left(d^{2}\rho /dx^{2}\right)}_{\max}=\max\left\{\left|d^{2}f/dx^{2}\right|,\left|d^{2}g/dx^{2}\right|\right\}$$

has the same fluctuating tendency as the gradient d*ρ*/d*x* in Table 8 (although its variation is consistent with the sintering temperature for pressed ceramics—Table 7). Therefore, both derivatives are not consistent with the variation of the sintering temperature, and cannot be used in the assessment. 

## 5. Conclusions

Pressed ceramic prosthetic constructs are one of the best aesthetic solutions for the dental rehabilitation of the patients, especially for the frontal zone. However, these structures are as a result of fractures and chipping, most of the time because of technological procedures. Not controlling the pressing temperature for the ceramic core represents an important cause for these issues; it is hard to evaluate them and to optimize the process in a common dental laboratory. The only method to evaluate the correspondence between the programmed and the real temperature in the oven has so far been, as pointed out in the Introduction, to fire supplemental samples made of the purest available ceramics and to assess their quality visually. Errors result in modifying the crystalline structure of the same ceramic material that looks different at different temperatures (normal, smaller, or higher than the temperature recommended by the manufacturers). These modifications could lead to fractures or chipping of the prosthetic construct. However, no direct optical methods or Rx evaluation can provide any information about such modifications.

To tackle this issue, the present study considered pressed integral ceramic dental prostheses sintered at a normal temperature (as prescribed by the manufacturer), as well as at lower and higher sintering temperatures than normal by 50 °C, which can be reached in the dental oven. An in-house swept source (SS) OCT imaging instrument enhanced with Master-Slave (MS) technology [31] was used to directly provide en-face OCT images (i.e., delivered in real time and not inferred by slicing volumetric reconstructions, therefore readily available to the dental technician). Using the data produced with this instrument, the loss of calibration of the sintering oven was studied. The present study demonstrated that such en-face OCT images can provide rules-of-thumbs for the qualitative assessment of the loss of calibration of ceramic ovens (while B-scans can only point out defects and interface issues between ceramic layers). 

The different tendencies identified for the three sample groups considered also allowed for a quantitative assessment of the above issue by obtaining the reflectivity profiles, their average graphs, and their corresponding functions derived analytically. The data from this study, as well as from a previous study [11], on metal ceramics prostheses (with a different ceramic material) were used to study which is the most appropriate parameter to be utilized in such assessments. A set of characteristic parameters was obtained from the analytic functions, for both ceramics. We demonstrated that the ratio of the maximum and minimum (average) reflectivity is the best (i.e., the most consistent parameter) to characterize the sintering temperature variations in the dental oven.

Also, an interesting conclusion was obtained, namely: the reflectivity profiles obtained at the normal sintering temperature can serve as a quality indicator when comparing different ceramic materials. Although the reflectivity profiles and (average) graphs are different for every sample (and ceramics), the methodology developed can be applied to any dental ceramic sample to assess the issues of the granulation. To our knowledge, this is the first time such a quantitative approach has been made.

From the clinical point of view, a simple OCT evaluation made each day (with a procedure that could take only a few minutes) could monitor the temperature variation in the dental oven. The necessity of a recalibration process can thus be determined. The simple and fast OCT-based assessment method developed can decide if the quality of the prosthetic construct is suitable for the oral environment, or if it is recommended to be remade and to send it to the clinical office. In the context of the enhancement of the applications (thus, of the availability) of OCT for dental medicine, such a method could become common practice in the near future. 

Future work: this direction of study includes considering other ceramic materials to determine their behavior from the point of view of sintering temperatures, to compare the behavior of different materials (and dental constructs), and for further analytical modeling of these aspects.

## Figures and Tables

**Table 1:** Modeling of the reflection functions: Group L—dental pressed ceramics.

*x*	$\left[0,x_{m}\right)=\left[0,1.5\right)$	$\left[x_{m},x_{\max}\right)=\left[1.5,3.25\right)$
*ρ*(*x*)	$f(x)=\frac{\left(\rho_{0}-\rho_{\min}\right)}{x_{m}}\left(\frac{x^{2}}{x_{m}}-2x\right)+\rho_{0}$	$\begin{array}{l} g(x)=\frac{\rho_{\max}-\rho_{\min}}{{\left(x_{\max}-x_{m}\right)}^{2}}x\left(x-2x_{m}\right)+\\ +\frac{\rho_{\min}\left(x_{\max}-2x_{m}\right)x_{\max}-\rho_{\max}x_{m}^{2}}{{\left(x_{\max}-x_{m}\right)}^{2}} \end{array}$
	$f(x)=11.11x^{2}-33.33x+60$	$g(x)=15.35x^{2}-16.33x+69.53$
d*ρ*/d*x*	$f^{\prime }(x)=2\frac{\left(\rho_{0}-\rho_{\min}\right)}{x_{m}}\left(\frac{x}{x_{m}}-1\right)$	$g^{\prime }(x)=2\frac{\rho_{\max}-\rho_{\min}}{{\left(x_{\max}-x_{m}\right)}^{2}}\left(x-x_{m}\right)$
	$f^{\prime }(x)=22.22\cdot x-33.33$	$g^{\prime }(x)=30.7\cdot x-16.33$
d^2^*ρ*/d*x*^2^	$f^{\prime \prime }(x)=2\frac{\left(\rho_{0}-\rho_{\min}\right)}{x_{m}^{2}}=22.22\;{\mathrm{mm}}^{-2}$	$g^{\prime \prime }(x)=2\frac{\rho_{\max}-\rho_{\min}}{{\left(x_{\max}-x_{m}\right)}^{2}}=30.7\;{\mathrm{mm}}^{-2}$

**Table 2:** Modeling of the reflection functions: Group N—dental pressed ceramics.

*x*	$\left[0,x_{i}\right)=\left[0,2\right)$	$\left[x_{i},x_{\max}\right)=\left[2,3.25\right)$
*ρ*(*x*)	$f(x)=\frac{\rho_{\max}-\rho_{0}}{x_{M}}\left(-\frac{x^{2}}{x_{M}}+2x\right)+\rho_{0}$	$\begin{array}{l} g(x)=\frac{\rho_{i}-\rho_{0}}{{\left(x_{\max}-x_{i}\right)}^{2}}x\left(x-2x_{\max}\right)+\\ +\frac{\rho_{i}x_{\max}^{2}-\rho_{0}\left(2x_{\max}-x_{i}\right)x_{i}}{{\left(x_{\max}-x_{i}\right)}^{2}} \end{array}$
	$f(x)=-9.6x^{2}+24x+60$	$g(x)=6.4x^{2}-41.6x+127.6$
d*ρ*/d*x*	$f^{\prime }(x)=2\frac{\rho_{\max}-\rho_{0}}{x_{m}}\left(-\frac{x}{x_{M}}+1\right)$	$g^{\prime }(x)=2\frac{\rho_{i}-\rho_{0}}{{\left(x_{\max}-x_{i}\right)}^{2}}\left(x-x_{\max}\right)$
	$f^{\prime }(x)=-19.2\cdot x+24$	$g^{\prime }(x)=12.8\cdot x-41.6$
d^2^*ρ*/d*x*^2^	$f^{\prime \prime }(x)=-2\frac{\left(\rho_{\max}-\rho_{0}\right)}{x_{m}^{2}}=-19.2\;{\mathrm{mm}}^{-2}$	$g^{\prime \prime }(x)=2\frac{\rho_{i}-\rho_{0}}{{\left(x_{\max}-x_{i}\right)}^{2}}=12.8\;{\mathrm{mm}}^{-2}$

**Table 3:** Modeling of the reflection functions: Group H—dental pressed ceramics.

*x*	$\left[0,x_{i}=2x_{M}\right)=\left[0,1.4\right)$	$\left[x_{i},x_{\max}\right)=\left[1.4,3.25\right)$
*ρ*(*x*)	$f(x)=\frac{\rho_{\max}-\rho_{0}}{x_{M}}\left(-\frac{x^{2}}{x_{M}}+2x\right)+\rho_{0}$	$\begin{array}{l} g(x)=\frac{\rho_{0}-\rho_{\min}}{{\left(2x_{M}-x_{m}\right)}^{2}}x\left(x-2x_{m}\right)+\\ +\frac{\rho_{0}x_{m}^{2}+4\rho_{\min}\left(2x_{m}-x_{M}\right)x_{M}}{{\left(2x_{M}-x_{m}\right)}^{2}} \end{array}$
	$f(x)=30.61x^{2}-42.85x+60$	$g(x)=23.12x^{2}-107.76x+170.5$
d*ρ*/d*x*	$f^{\prime }(x)=2\frac{\rho_{\max}-\rho_{0}}{x_{m}}\left(-\frac{x}{x_{M}}+1\right)$	$g^{\prime }(x)=2\frac{\rho_{o}-\rho_{\min}}{{\left(2x_{M}-x_{m}\right)}^{2}}\left(x-x_{m}\right)$
	$f^{\prime }(x)=61.22\cdot x-42.85$	$g^{\prime }(x)=46.24\cdot x-107.76$
d^2^*ρ*/d*x*^2^	$f^{\prime \prime }(x)=-2\frac{\left(\rho_{\max}-\rho_{0}\right)}{x_{m}x_{M}}=30.61\;{\mathrm{mm}}^{-2}$	$g^{\prime \prime }(x)=2\frac{\rho_{0}-\rho_{\min}}{{\left(2x_{M}-x_{m}\right)}^{2}}=46.24\;{\mathrm{mm}}^{-2}$

**Table 4:** Modeling of the reflection functions: Group L100—metal ceramic prostheses.

*x*	$\left[0,x_{i}\right)=\left[0,1\right)$	$\left[x_{i},x_{\max}\right)=\left[1,3.25\right)$
*ρ*(*x*)	$f(x)=\frac{\rho_{i}-\rho_{0}}{x_{i}^{2}}x^{2}+\rho_{0}$	$\begin{array}{l} g(x)=\frac{4(\rho_{i}-\rho_{\min})x(x-x_{i}-x_{\max})}{{\left(x_{\max}-x_{i}\right)}^{2}}+\\ +\frac{\rho_{i}{\left(x_{i}+x_{\max}\right)}^{2}-4\rho_{\min}x_{i}x_{\max}}{{\left(2x_{M}-x_{m}\right)}^{2}} \end{array}$
	$f(x)=3x^{2}+27$	$g(x)=2.37x^{2}-10.07x+361.25$
d*ρ*/d*x*	$f(x)=2\frac{\rho_{i}-\rho_{0}}{x_{i}^{2}}x$	$g^{\prime }(x)=2\frac{\rho_{o}-\rho_{\min}}{{\left(2x_{M}-x_{m}\right)}^{2}}\left(x-x_{m}\right)$
	$f^{\prime }(x)=6x$	$g^{\prime }(x)=4.74\cdot x-10.07$
d^2^*ρ*/d*x*^2^	$f^{\prime \prime }(x)=6\;{\mathrm{mm}}^{-2}$	$g^{\prime \prime }(x)=2\frac{\rho_{0}-\rho_{\min}}{{\left(2x_{M}-x_{m}\right)}^{2}}=2.37\;{\mathrm{mm}}^{-2}$

**Table 5:** Modeling of the reflection functions: Group L30—metal ceramic prostheses.

*x*	$\left[0,x_{i}\right)=\left[0,1\right)$	$\left[x_{i},x_{\max}\right)=\left[1,3.25\right)$
*ρ*(*x*)	$f(x)=\frac{\rho_{i}-\rho_{0}}{x_{i}^{2}}x^{2}+\rho_{0}$	$g(x)=\rho_{0}$
	$f(x)=3x^{2}+27$	g(x)=25
d*ρ*/d*x*	$f^{\prime }(x)=2\frac{\rho_{i}-\rho_{0}}{x_{i}^{2}}x$	$g^{\prime }(x)=0$
	$f^{\prime }(x)=6x$	
d^2^*ρ*/d*x*^2^	$f^{\prime \prime }(x)=6\;{\mathrm{mm}}^{-2}$	$g^{\prime \prime }(x)=0$

**Table 6:** Modeling of the reflection functions: Group H30—metal ceramic prostheses.

*x*	$\left[0,\frac{x_{\max}}{3}\right)=\left[0,1.08\right)$	$\left[\frac{x_{\max}}{3},\frac{2x_{\max}}{3}\right)=\left[1.08,2.17\right)$	$\left[\frac{2x_{\max}}{3},x_{\max}\right)=\left[2.17,3.25\right)$
*ρ*(*x*)	$f(x)=\frac{12A}{x_{\max}^{2}}x\left(3x-x_{\max}\right)+\rho_{0}$	$g(x)=\frac{36A}{x_{\max}^{2}}x\left(x-x_{\max}\right)+\rho_{0}+10A$	$h(x)=\frac{6A}{x_{\max}^{2}}x\left(3x-10x_{\max}\right)+\rho_{0}+24A$
	$f(x)=10.22x^{2}-11.08x+31$	$g(x)=-10.22x^{2}-10.22x+61$	$h(x)=10.22x^{2}+55.38x+55.3$
d*ρ*/d*x*	$f^{\prime }(x)=20.44x-11.08$	$g^{\prime }(x)=-20.44x-10.22$	$h^{\prime }(x)=20.44x+55.38$
d^2^*ρ*/d*x*^2^	$f^{\prime \prime }(x)=20.44\;{\mathrm{mm}}^{-2}$	$g^{\prime \prime }(x)=-20.44\;{\mathrm{mm}}^{-2}$	$h^{\prime \prime }(x)=20.44\;{\mathrm{mm}}^{-2}$
The notation $\rho_{0}-\rho_{\min}=\rho_{\max}-\rho_{0}=A$ in Figure 8(b2) was used.			

**Table 7:** Parameters of the reflectivity profiles, graphs, and functions—for pressed ceramic dental prostheses.

Parameter (Figure 6)	Group L	Group N	Group H
(ρ_min_)_peak_	34	58	41
(ρ_max_)_peak_	84	91	89
Δρ_peaks_ = (ρ_max_)_peak_ − (ρ_min_)_peak_	50	33	48
k_peaks_ = (ρ_max_)_peak_/(ρ_min_)_peak_	2.47	1.57	2.17
ρ_min_	35	60	41
ρ_max_	82	85	89
Δρ = ρ_max_ − ρ_min_	47	15	48
k = ρ_max_/ρ_min_	2.34	1.42	2.17
(dρ/dx)_max_ = max{|f′(x)|, |g′(x)|}	53.71	24	42.86
(d^2^ρ/dx^2^)_max_ = max{|f″(x)|, |g″(x)|}	30.69	19.2	61.22

**Table 8:** Parameters of the reflectivity profiles, graphs, and functions—for metal ceramic dental prostheses.

Parameter (Figure 7 and Figure 8)	Group L100	Group L30	Group N	Group H30	Group H50
(ρ_min_)_peak_	16	27	21	26	19
(ρ_max_)_peak_	28	34	32	38	34
Δρ_peaks_ = (ρ_max_)_peak_ − (ρ_min_)_peak_	12	7	11	12	15
k_peaks_ = (ρ_max_)_peak_/(ρ_min_)_peak_	1.75	2	1.52	1.46	1.79
ρ_min_	17	25	25	26	19
ρ_max_	27	32	25	43	34
Δρ = ρ_max_ − ρ_min_	10	7	0	17	15
k = ρ_max_/ρ_min_	1.59	1.28	1	1.65	1.79
(dρ/dx)_max_ = max{|f′(x)|, |g′(x)|}	14.81	10	0	11.08	2.77
(d^2^ρ/dx^2^)_max_ = max{|f″(x)|, |g″(x)|}	14	10	0	20.44	0

## Appendix A

### Appendix A.1. Dental Pressed Ceramics: Modeling of the Reflection Curves

#### Appendix A.1.1. Group L: Dental Pressed Ceramics

A parabolic function is considered for each portion of the *ρ*(*x*) curve in Figure 6(a2); for the first portion, one has the following:

(A1) $$f(x)=ax^{2}+bx+c\;\mathrm{and}\;f^{\prime }(x)=2ax+b,\;\mathrm{where}\;a,b,c=\mathrm{constant}$$

Constants *a*, *b*, and *c* are obtained by imposing the conditions extracted from Figure 6(a2), as follows:

(A2) $$-b/2a=x_{m};\;-\Delta /4a=\rho_{\min};\;f(0)=\rho_{0}$$

where $\Delta =b^{2}-4ac$. With Equation (A1) in (A2), on has the following:

(A3) $$a=\left(\rho_{0}-\rho_{\min}\right)/x_{m}^{2};\;b=-2\left(\rho_{0}-\rho_{\min}\right)/x_{m};\;c=\rho_{0}$$

Using the parameters in Figure 6(a2), one has the following: *a* = 11.11 mm^−2^; *b* = −33.33 mm^−1^; *c* = 60.

Another parabolic function is considered for the second portion of the *ρ*(*x*) curve (Figure 6(a2)), as follows:

(A4) $$g(x)=ax^{2}+bx+c\;\mathrm{and}\;g^{\prime }(x)=2ax+b,\;\mathrm{where}\;a,b,c=\mathrm{constant}$$

Constants *a*, *b*, and *c* of this second function are obtained by imposing the second set of conditions from Figure 6(a2), as follows:

(A5) $$-b/2a=x_{m};\;-\Delta /4a=\rho_{\min};\;g(x_{\max})=\rho_{\max}$$

With Equation (A5) in (A4), one has the following:

(A6) $$a=\frac{\rho_{\max}-\rho_{\min}}{{\left(x_{\max}-x_{m}\right)}^{2}};\;b=-2\frac{\rho_{\max}-\rho_{\min}}{{\left(x_{\max}-x_{m}\right)}^{2}}x_{m};\;c=\frac{\rho_{\min}\left(x_{\max}-2x_{m}\right)x_{\max}-\rho_{\max}x_{m}^{2}}{{\left(x_{\max}-x_{m}\right)}^{2}}$$

Using the parameters in Figure 5(a2), *a* = 15.35 mm^−2^; *b* = −16.33 mm^−1^; *c* = 69.53.

Functions f(*x*) and g(*x*) of the *ρ*(*x*) curve are provided in Table 1, as well as gradients f′(*x*) and g′(*x*), and second derivatives f″(*x*) and g″(*x*). The gradients are also shown in Figure 6(a3).

#### Appendix A.1.2. Group N: Dental Pressed Ceramics

The first portion of the *ρ*(*x*) curve—Figure 6(b2)—has a parabolic function similar to Equation (A1). Constants *a*, *b*, and *c* are obtained using the conditions from Figure 6(b2), as follows:

(A7) $$-b/2a=x_{M};\;-\Delta /4a=\rho_{\max};\;f(0)=\rho_{0}$$

therefore, with Equation (A1), one has the following:

(A8) $$a=-\left(\rho_{\max}-\rho_{0}\right)/x_{M}^{2};\;b=2\left(\rho_{\max}-\rho_{0}\right)/x_{M};\;c=\rho_{0}$$

Using the parameters in Figure 6(b2), one has the following: *a* = −9.6 mm^2^; *b* = 24 mm; *c* = 60. The value of the function in *x*_i_ is also obtained, as follows:

(A9) $$\rho_{i}=f(x_{i})=\frac{\left(\rho_{\max}-\rho_{\min})(2x_{M}-x_{i}\right)}{x_{M}^{2}}+\rho_{0}$$

and with the values in Figure 6(b3), *ρ*_i_ ≈ 70.

For the second portion of the *ρ*(*x*) curve in Figure 6(b2), Equation (A4) is used, with the following conditions:

(A10) $$-b/2a=x_{\max};\;-\Delta /4a=\rho_{0};\;g(x_{i})=\rho_{i}$$

from which constants *a*, *b*, and *c* of this second function are obtained, as follows:

(A11) $$a=\frac{\rho_{i}-\rho_{0}}{{\left(x_{\max}-x_{i}\right)}^{2}};\;b=-2\frac{\rho_{i}-\rho_{0}}{{\left(x_{\max}-x_{i}\right)}^{2}}x_{\max};\;c=\frac{\rho_{i}x_{\max}^{2}-\rho_{0}x_{i}(2x_{\max}-x_{i})}{{\left(x_{\max}-x_{i}\right)}^{2}}$$

Using the parameters in Figure 6(b2), one has the following: *a* = 6.4 mm^−2^; *b* = −41.6 mm^−1^; *c* = 127.6.

Functions f(*x*) and g(*x*) of the *ρ*(*x*) curve are provided in Table 2, as well as gradients f′(*x*) and g′(*x*), and the second derivatives f″(*x*) and g″(*x*). The gradients are also shown in Figure 6(b3).

#### Appendix A.1.3. Group H: Dental Pressed Ceramics

It can be seen from the profile of the *ρ*(*x*) curve in Figure 6(c2), that its first portion has a parabolic function similar to Equation (A1), with constants *a*, *b*, and *c* given exactly by Equation (A8), as the conditions (A7) are the same. The values of the constants are different; using the parameters in Figure 6(c2), one has the following: *a* = −30.61 mm^−2^; *b* = 42.85 mm^−1^; *c* = 60. Also, from Figure 6(c2), f(0) = *ρ*_0_ = f(*x*_i_) and f′(0) = −f′(*x*_i_) = 42.85 mm^−1^.

For the second portion of the *ρ*(*x*) curve in Figure 6(c2), Equation (A4) is used, with the following conditions:

(A12) $$-b/2a=x_{m};\;-\Delta /4a=\rho_{\min};\;g(2x_{M})=\rho_{0};\;g(x_{\max})=\rho (x_{\max})$$

from which constants *a*, *b*, and *c* of this second function have the following expressions:

(A13) $$a=\frac{\rho_{0}-\rho_{\min}}{{\left(2x_{M}-x_{m}\right)}^{2}};\;b=-2\frac{\rho_{0}-\rho_{\min}}{{\left(2x_{M}-x_{m}\right)}^{2}}x_{m};\;c=\frac{\rho_{0}x_{m}^{2}-4\rho_{\min}\left(x_{m}-x_{M}\right)x_{M}}{{\left(2x_{M}-x_{m}\right)}^{2}}$$

The issue is to determine *x*_m_, which is done from the supplemental condition, as follows:

(A14) $$g^{\prime }(2x_{M})=f^{\prime }(2x_{M})\;=>\;-2\frac{\rho_{0}-\rho_{\min}}{x_{m}-2x_{M}}x_{m}=-2\frac{\rho_{\max}-\rho_{0}}{x_{M}}\;=>\;x_{m}=\frac{2\rho_{\max}-\rho_{\min}-\rho_{0}}{\rho_{\max}-\rho_{0}}x_{M}$$

from which *x*_m_ = 2.33 mm. Using this value, the parameters in Figure 6(b3), and Equation (A13), one has the following: *a* = 23.12 mm^−2^; *b* = −107.76 mm^−1^; *c* = 170.5. The exact value of g(*x*_max_) is also obtained, that is, 64.57.

Functions f(*x*) and g(*x*) of the *ρ*(*x*) curve are provided in Table 3, as well as gradients f′(*x*) and g′(*x*), and second derivatives f″(*x*) and g″(*x*). The gradients are also shown in Figure 6(c3).

## Appendix B

### Appendix B.1. Metal Ceramic Prostheses: Modeling of the Reflection Curves

#### Appendix B.1.1. Group L100: Metal Ceramic Prostheses

The first portion of this group’s curve *ρ*(*x*)—Figure 7(a2)—has a parabolic function, given by Equation (A1). Constants *a*, *b*, and *c* are obtained using the conditions from Figure 7(a2), as follows:

(A15) $$-b/2a=x_{M};\;-\Delta /4a=\rho_{0};\;f(x_{i})=\rho_{i}$$

therefore, with Equation (A1), one has the following:

(A16) $$a=\left(\rho_{i}-\rho_{0}\right)/x_{i}^{2};\;b=0;\;c=\rho_{0}$$

Using the parameters in Figure 7(a2), one has the following: *a* = 3 mm^−2^; *b* = 0; *c* = 27.

For the second portion of the *ρ*(*x*) curve in Figure 7(a2), Equation (A4) is used, with the following conditions:

(A17) $$-b/2a=(x_{i}+x_{\max})/2=x_{m};\;-\Delta /4a=\rho_{\min};\;g(x_{i})=g(x_{\max})=\rho_{i}$$

from which constants *a*, *b*, and *c* of this second function are obtained, as follows:

(A18) $$a=\frac{4(\rho_{i}-\rho_{\min})}{{\left(x_{\max}-x_{i}\right)}^{2}};\;b=-4\frac{\rho_{i}-\rho_{\min}}{{\left(x_{\max}-x_{i}\right)}^{2}}\left(x_{\max}+x_{i}\right);\;c=\frac{\rho_{i}{\left(x_{\max}+x_{i}\right)}^{2}-4\rho_{\min}x_{i}x_{\max}}{{\left(x_{\max}-x_{i}\right)}^{2}}$$

Using the parameters in Figure 7(a2), one has the following: *a* = 2.37 mm^−2^, *b* = −10.07 mm^−1^, *c* = 27.7, while g(*x*_i_) = g(*x*_max_) = −0.59 mm^−1^.

Functions f(*x*) and g(*x*) of the *ρ*(*x*) curve are provided in Table 4, as well as gradients f′(*x*) and g′(*x*), and second derivatives f″(*x*) and g″(*x*). The gradients are also shown in Figure 7(a3).

#### Appendix B.1.2. Group L30: Metal Ceramic Prostheses

From the *ρ*(*x*) curve in Figure 7(b2), the first portion has a parabolic function similar to Equation (A1), with constants *a*, *b*, and *c* given exactly by Equation (A8), as the conditions (A7) are the same (Table 5). The values of the constants are different; using the parameters in Figure 7(b2), one has the following: *a* = −5 mm^−2^; *b* = 10 mm^−1^; *c* = 25. Also using Figure 7(b2), one has the following: f(0) = *ρ*_0_ and f′(0) = −f′(2*x*_M_) = 10 mm^−1^.

#### Appendix B.1.3. Group H30: Metal Ceramic Prostheses

This group’s curve *ρ*(*x*)—Figure 8(b2)—has three parabolic portions and functions.

For $x\in \left[0,x_{\max}/3\right)$, using Equation (A1), constants *a*, *b*, and *c* of the first parabolic function f(*x*) are obtained using the conditions from Figure 8(b2), as follows:

(A19) $$-b/2a=x_{\max}/6;\;-\Delta /4a=\rho_{\min};\;f(0)=\rho_{0}=f(x_{\max}/3)$$

therefore, with a convenient notation $\rho_{0}-\rho_{\min}=\rho_{\max}-\rho_{0}=A$, one has the following:

(A20) $$a=36A/x_{\max}^{2};\;b=-12A/x_{\max};\;c=\rho_{0}.$$

Using the parameters in Figure 8(b2), one has the following: *a* = 10.22 mm^−2^; *b* = −11.08; *c* = 31.

For $x\in \left[x_{\max}/3,2x_{\max}/3\right)$, the second portion g(*x*) of the *ρ*(*x*) curve in Figure 8(b2) has the following conditions:

(A21) $$-b/2a=x_{\max}/2;\;-\Delta /4a=\rho_{\max};\;g(x_{\max}/3)=\rho_{0}$$

from which constants *a*, *b*, and *c* of this second function are obtained, as follows:

(A22) $$a=-36A/x_{\max}^{2};\;b=36A/x_{\max};\;c=\rho_{0}+10A$$

Using the parameters in Figure 8(b2), one has the following for this function: *a* = −10.22 mm^−2^, *b* = 10.22 mm^−1^, *c* = 61.

For $x\in \left[2x_{\max}/3,x_{\max}\right)$, the third parabolic portion h(*x*) of the *ρ*(*x*) curve in Figure 8(b2), has the following conditions:

(A23) $$-b/2a=5x_{\max}/6;\;-\Delta /4a=\rho_{\min};\;h(x_{\max})=\rho_{0}$$

from which constants *a*, *b*, and *c* of this second function are obtained, as follows:

(A24) $$a=36A/x_{\max}^{2};\;b=-60A/x_{\max};\;c=\rho_{0}+24A$$

Using the parameters in Figure 8(b2), one has the following for this function: *a* = 10.22 mm^−2^, *b* = 55.38 mm^−1^, *c* = 55.3.

Functions f(*x*) and g(*x*) of the *ρ*(*x*) curve are provided in Table 6, as well as gradients f′(*x*) and g′(*x*), and second derivatives f″(*x*) and g″(*x*). The gradients are also shown in Figure 8(b3).
